# Temporal malleability to auditory feedback perturbation is modulated by rhythmic abilities and auditory acuity

**DOI:** 10.3389/fnhum.2022.885074

**Published:** 2022-09-15

**Authors:** Miriam Oschkinat, Philip Hoole, Simone Falk, Simone Dalla Bella

**Affiliations:** ^1^Institute of Phonetics and Speech Processing, Ludwig Maximilian University of Munich, Munich, Germany; ^2^International Laboratory for Brain, Music and Sound Research, Montreal, QC, Canada; ^3^Department of Linguistics and Translation, University of Montreal, Montreal, QC, Canada; ^4^Centre for Research on Brain, Language and Music, Montreal, QC, Canada; ^5^Department of Psychology, University of Montreal, Montreal, QC, Canada; ^6^University of Economics and Human Sciences in Warsaw, Warsaw, Poland

**Keywords:** temporal auditory feedback perturbation, feedforward malleability, auditory acuity, finger tapping, rhythmic abilities

## Abstract

Auditory feedback perturbation studies have indicated a link between feedback and feedforward mechanisms in speech production when participants compensate for applied shifts. In spectral perturbation studies, speakers with a higher perceptual auditory acuity typically compensate more than individuals with lower acuity. However, the reaction to feedback perturbation is unlikely to be merely a matter of perceptual acuity but also affected by the prediction and production of precise motor action. This interplay between prediction, perception, and motor execution seems to be crucial for the timing of speech and non-speech motor actions. In this study, to examine the relationship between the responses to temporally perturbed auditory feedback and rhythmic abilities, we tested 45 adult speakers on the one hand with a temporal auditory feedback perturbation paradigm, and on the other hand with rhythm perception and production tasks. The perturbation tasks temporally stretched and compressed segments (onset + vowel or vowel + coda) in fluent speech in real-time. This technique sheds light on the temporal representation and the production flexibility of timing mechanisms in fluent speech with respect to the structure of the syllable. The perception tasks contained staircase paradigms capturing duration discrimination abilities and beat-alignment judgments. The rhythm production tasks consisted of finger tapping tasks taken from the BAASTA tapping battery and additional speech tapping tasks. We found that both auditory acuity and motor stability in finger tapping affected responses to temporal auditory feedback perturbation. In general, speakers with higher auditory acuity and higher motor variability compensated more. However, we observed a different weighting of auditory acuity and motor stability dependent on the prosodic structure of the perturbed sequence and the nature of the response as purely online or adaptive. These findings shed light on the interplay of phonological structure with feedback and feedforward integration for timing mechanisms in speech.

## Introduction

In speech production, speakers execute speech movements based on learned internal representations (feedforward system) while the sensory experience of the produced outcome, such as auditory or somatosensory feedback, serves to monitor and evaluate the process constantly (feedback system). The interaction of feedback and feedforward systems in speech production has been of significant interest in speech research and has mainly been probed with real-time auditory feedback perturbations. In auditory feedback perturbation paradigms, speakers hear their voice over headphones while one or more parameters in the acoustic signal are altered in (almost) real-time. In response, speakers were found to counteract the applied feedback shift (*compensate*) in production. Compensation was classified as purely an *online response* when adjustments occurred ∼120–200 ms after perturbation onset in the ongoing production process (Burnett et al., 1998; Purcell and Munhall, 2006). When speakers compensated in future productions of the same/similar unperturbed speech segments, they were said to *adapt*. Online responses hereby support the incorporation of auditory feedback into the control level, adaptation indicates an update of the underlying motor plan for the respective production. While auditory feedback perturbations in the spectral domain (e.g., formant or pitch shifts) have been extensively studied, a few recent studies have started to investigate the role of auditory feedback for speech *timing* (e.g., Cai et al., 2011; Mitsuya et al., 2014; Floegel et al., 2020; Oschkinat and Hoole, 2020, 2022; Karlin et al., 2021). Analogously to spectral feedback perturbations, the majority of speakers was found to compensate for temporal feedback shifts both in the online control as well as in future productions (adaptation), supporting the incorporation of auditory feedback into speech timing mechanisms on control and planning levels. However, there are crucial differences between responses to spectral and temporal auditory feedback perturbations. In spectral perturbations, online responses to altered feedback could be observed in either direction (e.g., with an increase or decrease of formant frequencies in production). For the temporal domain, this bidirectionality is not given naturally: While it is perhaps possible to lengthen a sequence in production that was perceived shorter (more specifically, terminated early) or react to a delay in the auditory feedback, it is not possible to shorten segments online as a reaction to longer percepts (as the sequence is already terminated in production when the auditory stretch is received). Therefore, every shortening response as a reaction to a stretched speech signal must be adaptive. Further, responses in reaction to the perturbation that are not necessarily compensatory and not at the perturbation site itself (e.g., lengthening of following segments as a reaction to a preceding stretched segment) were classified as *reactive feedback control.* These effects might aim at recovering relative durations within a higher prosodic timeframe (e.g., adjusting segment proportions within a syllable) or can be rather unspecific responses to a disturbance in the feedback that demands attention and time to process. Reactive feedback responses seem similar to responses elicited by generally delayed auditory feedback, where speakers were found to slow down their speech rate or prolong speech elements in response (Yates, 1963).

In our previous study, Oschkinat and Hoole (2020), response patterns differed dependent on the part of the syllable that experienced the temporal shift. While speakers compensated and, in some cases, adapted for a temporally manipulated nucleus and coda of a syllable, no significant effect was found for temporally stretched syllable onsets. The subsequent studies by Karlin et al. (2021) and Oschkinat and Hoole (2022) produced similar results regarding the responses: Speakers adjusted their productions (in absolute segment durations) for perturbed nuclei and codas, but not for onsets. We suggested that, at least for timing relations in speech, the prosodic structure of the syllable causes segments to be more or less malleable in their articulatory execution than others. This hypothesis was based on insights into the articulatory structure of the syllable elaborated in the Articulatory Phonology/Task-Dynamics framework. In modeling inter-gestural timing, the syllable segments are modeled as coupled oscillators with different coordinative relations. In some languages, such as English or German, gestures couple mainly in-phase or anti-phase with the adjacent gestures, dependent on syllable position. Thereby, in syllable onsets, consonant gestures are coupled anti-phase with each other but in-phase with the following vowel, while in codas each gesture is coupled locally anti-phased with the preceding one. The more global coupling of onsets with the vowel constitutes a greater temporal/articulatory stability than the local anti-phase coupling of the coda segment with the vowel (Byrd, 1996; Browman and Goldstein, 2000; Goldstein and Pouplier, 2014). For detailed consideration of the evidence for differential coordination patterns related to syllable position specifically for German see Pouplier (2012). Hence, codas should be more malleable when it comes to an auditory perturbation of timing than the more articulatorily entrenched onset patterns. In our follow-up study, Oschkinat and Hoole (2022), differences in the response patterns were not only observed for different parts of the syllable, but also for syllables with different stress patterns and syllable position within the word. Both our previous studies (Oschkinat and Hoole, 2020, 2022) indicated that auditory feedback can be used for temporal corrections in the speech production process, but that prosodic structure of the perturbed segment plays a role. With regard to current speech production models, these findings support the incorporation of auditory feedback into the speech production process as modeled in the Directions into Velocities of Articulators model (DIVA model, Guenther, 2006) but for *speech timing*, combined with knowledge about the prosodic stability of segments (as elaborated in Articulatory Phonology/Task-Dynamics; cf. Browman and Goldstein, 1992).

The role of perception and the feedback system for speech acquisition and speech production has been considered crucially relevant. According to the DIVA model, speakers rely on spatio-temporal representations of speech elements (speech targets) in feedforward control. These speech targets are established via auditory and somatosensory feedback in speech acquisition (Guenther, 2016). Thereby, the size of the size of an acquired speech target is assumed to depend on individual auditory acuity and sensory error detection performance. Speakers with better auditory acuity establish smaller speech targets, resulting in more distinct productions of different speech sounds and less variability in production (Perkell et al., 2004a,b, 2008; Ghosh et al., 2010). Individual differences in auditory acuity became a further focus of interest in connection with auditory feedback perturbation studies. Villacorta et al. (2007) assessed auditory acuity in the discrimination of the first formant (F1) in vowels and set it in relation to reactions to upward and downward shifts of F1 in the same vowels. They found that the better the individual auditory acuity, the more the speaker compensated for the applied feedback alteration. This conclusion was also drawn by Brunner et al. (2011) for perturbed consonants. They found speakers with a higher auditory acuity to produce /s/ and /∫/ with a more distinct acoustical contrast and to use compensation strategies to a greater extent than low acuity speakers.

While individual abilities in feedback control have been considered to be crucial influencing factors in building and controlling speech targets, much less attention has been given to the thought that also feedforward mechanisms, more precisely motor execution abilities, are governed by limits of individual abilities. The study by Martin et al. (2018) investigated relations between responses to spectral auditory feedback perturbations and feedback capacities (auditory acuity), as well as general cognitive control skills as an indicator for feedforward abilities. They found auditory acuity relevant for predicting responses, but not the executive control tasks. Apart from general cognitive abilities, another aspect that could plausibly influence distinctiveness in speech production is the ability to execute motor commands for desired speech targets precisely in time and space. However, the role of temporal precision in speech production is relatively understudied. Nevertheless, a rather different strand of research has investigated temporal precision and rhythmic abilities in non-speech motor execution. An indication for the relevance of internal timing abilities in feedforward control has been provided by research on rhythmic finger tapping with or without auditory stimuli (Repp, 2005; Repp and Su, 2013; Dalla Bella et al., 2017). In typical tapping tasks, participants tap regularly at a self-chosen rate (unpaced tapping), or along with an accompanying beat or sound sequence or synchronize to music (paced tapping, Dalla Bella et al., 2017). Unpaced tapping tasks give the examiner insight into feedforward timing mechanisms and their stability in motor execution (see Drake et al., 2000). Tapping to a beat, on the other hand, tests for sensorimotor synchronization (see Repp and Su, 2013 for an overview).

A link between non-verbal sensorimotor timing abilities and speech production was found when testing finger tapping performance in non-impaired speakers and speakers with speech timing disorders. For example, Falk et al. (2015) found weaker synchronization abilities with a metronome or a musical stimulus in children and adolescents who stutter than in non-stuttering peers. Individuals who stutter showed worse rhythmic tapping performance, with a tendency to over-anticipate the pacing events, than individuals who do not stutter. Another study tested for the connection of rhythmic variability in different motor domains in patients with Parkinson’s disease. Puyjarinet et al. (2019) found a link between rhythmic variability in paced finger tapping, variability in speech (oral diadochokinesis tasks), and variability in gait. They further found deficits in rhythm perception linked to deficits in rhythm production and concluded that rhythm impairments in different motor domains in patients with Parkinson’s disease might be caused by an impaired central rhythm mechanism (Puyjarinet et al., 2019). Further research on speech and non-speech timing showed that in speech with finger tapping, emphasis in one domain affects the other domain as well, e.g., stressing a syllable is accompanied by more emphasized tapping (Parrell et al., 2014).

Altogether, these studies point toward a strong link between motor behavior in speech and non-speech actions. This link is noteworthy in particular when investigating the role of feedforward stability for timing mechanisms in fluent speech. Indeed, it can be hypothesized that temporal stability in non-speech motor behavior is connected to temporal stability in speech motor control. Thereby, it has to be taken into consideration that, domain-independently, different motor timing tasks might require different underlying neural mechanisms. Neuroscientific research outlined different such mechanisms dependent on the demand of the timing task. Grube et al. (2010) and Teki et al. (2011, 2012) distinguished between event-based timing, which occurs relative to a beat, and duration-based timing, which requires the absolute estimation of temporal intervals, both mechanisms being associated with different brain regions (Teki et al., 2011). In speech production, it is assumable that different parts of an utterance or even of a syllable follow different timing strategies. The prediction of onsets in speech, for example, was suggested to be comparable with recurrences of a musical beat (Nozaradan et al., 2012; Peelle and Davis, 2012). Further, the supposed beat in an isochronous flow of speech syllables is located in the transition between onset and vowel (p-center, Morton et al., 1976). Accordingly, onset timing might be more closely related to event-based timing mechanisms. This assumption was supported by interpreting the brain regions involved in the timing mechanisms: Event-based timing was more associated with brain regions comprising the supplementary motor area and the premotor cortex (Teki et al., 2011). Both areas were found relevant for internal planning of motor movements within a precise timing plan rather than relying on sensory information. In our previous study (Oschkinat and Hoole, 2020), we assumed greater reliance on feedforward predictions in onsets leads to less compensation in auditory feedback perturbation. Nucleus and coda of the syllable might rather be timed with underlying duration-based timing mechanisms based on a word or syllable time frame.

The previous section outlined how perceptual abilities and general motor behavior connect to speech production. Further, the introduced timing mechanisms contribute to the complexity that is assumed to underlie the planning and control of speech timing.

The main goal of the following study is to shed light on the contribution of auditory feedback and motor timing abilities to speech production. Therefore, we examine the contribution of general internal timing stability as predictor for temporal speech feedforward stability, and the importance of feedback and feedforward mechanisms for executing and planning the temporal structure of fluent speech. To follow this aim, the present study assessed individual capacities in paced and unpaced finger tapping tasks and beat-based and duration-based perception tasks, and set them in relation to behavior during temporal auditory feedback perturbation from the data collected in our previous study (Oschkinat and Hoole, 2020). In doing so, we foreground the influence of individual auditory acuity and individual motor timing stability on speech production. Thereby, we address both feedback and feedforward systems as key actors for successful speech production. As for the outcome, we have two main hypotheses.

First, concerning the contribution of perception and motor execution, we expect speakers with better perceptual abilities (auditory acuity) to compensate more for temporal auditory feedback perturbations as found analogously for spectral properties of speech. This hypothesis is based on the idea that the better an auditory mismatch is perceived, the more (precisely) speakers can counteract it. Moreover, we expect speakers with a worse performance in motor execution in finger tapping tasks (speakers with a higher motor variability) to compensate more. This hypothesis ties up with the findings of Oschkinat and Hoole (2020, 2022), where a structurally less stable system was more malleable in the face of a temporal perturbation. We expect the effect of *structural* motor stability on timing behavior to extend to *individual* abilities in motor stability, which may also shape timing mechanisms in speech.

Second, regarding the nature of responses to the auditory feedback perturbation as an online response or adaptive, we expect to find perceptual acuity equally relevant for both online reactions and adaption, since both types of reaction require the ability to perceive the auditory mismatch and identify the direction for a compensatory response in the first place. General motor stability, on the other hand, should be a greater predictor for adaptation, since a less stable feedforward system should provide a greater tolerance toward updating the less stable representations. This hypothesis is tied to expectations about the relevance of auditory feedback and motor stability for different parts of the syllable, since the coda showed adaptation while the onset did not (Oschkinat and Hoole, 2020).

## Methods (procedure and data processing)

### Participants

Forty-five native speakers of German performed three testing blocks (described further below) in one testing session of approximately 2.5 h. Participants were between 19 and 30 years of age (mean age: 23 years, 34 females) and received financial compensation for their participation. Musicality was assessed with a questionnaire. Thirty-two participants stated they have received musical education on various instruments. Five of them reached a semi-professional level, indicating that they could earn money with music. Musicality was not a main focus of interest in the current study. However, additional analyses about effects of musicality on the response data can be found in the Appendix. None of the participants claimed to have any speech, voice, or hearing disorders. All of the participants started with the Auditory feedback perturbation block. After that, the order of the Tapping and Perception blocks was counterbalanced over participants. Participants were recruited in the Munich area and testing was approved by the ethics committee of the medical faculty of the Ludwig Maximilian University of Munich.

The following sections outline the three testing blocks Perturbation, Tapping, and Perception.

### Temporal auditory feedback perturbation

The perturbation response data was taken from the perturbation experiment reported in Oschkinat and Hoole (2020), including the same participants and their data. The following section briefly summarizes the procedure and measures of interest. For more thorough insight into the experiment, we point the reader to the original paper.

#### Setup

The temporal auditory feedback experiment tested the sensitivity to temporal perturbations with a special interest in position within the syllable. In two experiment conditions, the temporal structure of either onset + vowel (*Onset condition*) or vowel + coda (*Coda condition*) of the first syllable in a three-syllabic word was temporally altered (Onset condition: /′**pfa**nku:xən/, pancake; Coda condition: /′n**apf**ku:xən/, ring cake). Thereby, the first segment per condition was stretched in real-time (Onset condition: /pf/, Coda condition: /a/) and the following segment was compressed (Onset condition: /a/, Coda condition: /pf/) leading to an on-time signal after completion of both shifting directions. While this alteration results in perturbation being completely contained within a single syllable, it should be noted that the second segment of the perturbed part starts delayed by the amount of stretching of the first segment (plus the systems delay of approximately 25 ms that is needed for online manipulation).

Perturbations were achieved with the Audapter software package for formant and pitch shifts as well as time-warping developed by Cai et al. (2008, 2011) and Tourville et al. (2013). Participants received auditory feedback via E-A-RTone™ 3A in-ear earphones with foam eartips (3M, Saint Paul, MN, United States) and spoke into a Sennheiser H74 headset microphone (Wedemark, Germany) placed three cm from the corner of the mouth. The foam eartips ensure that the manipulated feedback rather than the airborne sound is predominantly perceived. In four blocks speakers uttered the phrase “besser Pfannkuchen” (Onset condition) or “besser Napfkuchen” (Coda condition). The carrier word “besser” (*better*) allowed for an online status tracking of the signal by the Audapter software to trigger the intended part within the target word (for more information on the online status tracking please refer to Oschkinat and Hoole, 2020).

**FIGURE 1:**
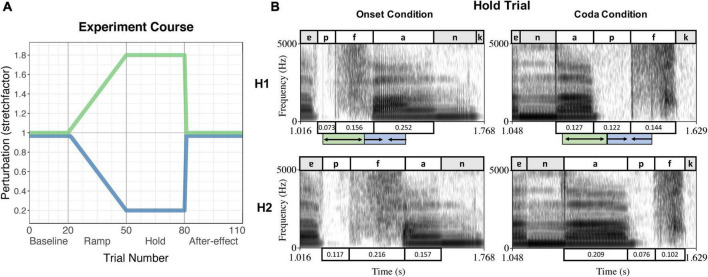
**(A)** Experiment course of the perturbation experiment, indicating the trial numbers and experiment phases on the *x*-axis and the stretchfactor of the perturbation on the *y*-axis. The green curve indicates the stretching of the first segment of the sequence, the blue curve indicates the compression of the second segment of the sequence. **(B)** Example of a Hold Trial per condition (Onset condition – left panel, Coda condition – right panel) produced by the same speaker. The upper panels show the spoken signal (H1), the lower panels the received perturbed auditory feedback (H2). Boxes above/underneath the spectrograms label the segments and their durations. The green/blue boxes below the upper panels indicate the stretching and compressing of the segments as triggered by the online status tracking, leading to the durations in the panels below (H2). Reproduced from Oschkinat and Hoole (2020), with the permission of the Acoustical Society of America.

While the online status tracking triggered the perturbation from the acoustic signal of each individual trial, the duration of the perturbation section (hence the duration of the onset + vowel or vowel + coda sequence) was determined manually by the experimenter (using Praat; Boersma and Weenink, 1999) for each participant in a pretest. This pretest included 15–20 trials of the experiment without perturbation, the number of trials depending on how fast the participant established a stable speech rate. The mean duration of the intended sequence over the pretest trials was then inserted into the protocol for testing. In the testing session, perturbation was applied in blocks: The first block consisted of 20 trials without perturbation (Baseline). In the second block, perturbation increased stepwise over 30 trials (Ramp phase) followed by 30 trials with maximum perturbation (Hold phase). After that, perturbation was abruptly removed and normal feedback restored for another 30 trials (After-effect phase). Figure 1A visualizes the applied perturbation over the course of the experiment, Figure 1B depicts spectrograms of the spoken signal (H1) and the received perturbation (H2) in both perturbation conditions during the Hold phase.

#### Analyses

The different perturbation phases allowed the examination of compensation as a general measure of reaction to the perturbation (Hold phase), and the classification of the response as either online control (when productions revert to the Baseline immediately in the After-effect phase) or adaptive (when adjustments remained into the After-effect phase where unaltered feedback is provided). For the purposes of the current study, the mean productions in the Hold phase as compared to the Baseline per participant are examined as an indicator for the strength of reaction during maximum perturbation. This measure will then be set in relation to measures from the tapping and the perception blocks. The analysis of the After-effect phase to classify responses as an online reaction or adaptive response was performed previously in Oschkinat and Hoole (2020) and will not be considered in detail in the current study. However, the determination of the nature of responses from this earlier analysis will turn out to be of substantial relevance for the interpretation and discussion of the present study further below.

For analyses, durations of the segments of interests were segmented manually in Praat. Production differences in word-normalized durations in the Hold phase (with maximum perturbation) relative to the Baseline for each segment of interest (CC /pf/ and V /a/) in each perturbation condition (Onset and Coda condition) were examined. Accordingly, four compensation measures are considered in the following calculations: Compensation to the onset segment in the Onset condition (Onset CC), compensation to the vowel in the Onset condition (Onset V), compensation to the vowel in the Coda condition (Coda V), and compensation to the coda segment in the Coda condition (Coda CC). These measures are the same as reported in Oschkinat and Hoole (2020) and will further be referred to as Onset CC, Onset V, Coda V, and Coda CC. The order represents the chronological appearance of the segments within the condition and consequently also represents the structure of the perturbation section, whereby the first segment per condition is stretched and the second segment is compressed.

**FIGURE 2:**
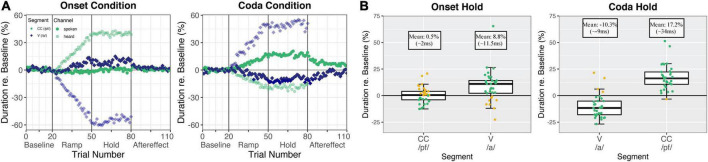
**(A)** Normalized relative durations averaged over all participants (*n* = 34 for the Onset condition, *n* = 33 for the Coda condition) per trial. The vowel /a/ is shown in blue rhombuses and CC /pf/ is shown in green round dots. The spoken signal is shown in solid colors and the perturbed (heard) signal is shown with higher transparency. The left panel visualizes the Onset condition and the right panel visualizes the Coda condition. **(B)** Normalized relative durations in the hold phase relative to the baseline mean (0) for vowel /a/ and CC /pf/ in the Onset condition (34 participants, left panel) and Coda condition (33 participants, right panel). Boxes correspond to the first and third quartiles and bars represent the median. Whiskers extend from the hinge to the highest/smallest value but no further than 1.5 interquartile range (IQR). Data beyond the whiskers are outliers. Individual participants are represented with colored dots where green dots mark compensatory behavior and golden dots mark a following of the perturbation direction. Reproduced from Oschkinat and Hoole (2020), with the permission of the Acoustical Society of America.

Figure 2A shows the produced and heard (perturbed) durations per segment and experiment condition over the course of the experiment. Figure 2B summarizes the production difference in the Hold phase as compared to baseline productions per segment of interest.

The perturbation data per participant and trial were scanned for correct triggering of the perturbation section at the intended part within the utterance. Since the online status tracking is based on previously determined intensity and duration thresholds, in some cases due to inter- and intra-speaker variability in speaking rate and style, the perturbation did not cover the intended speech sequence. Single trials were removed from calculations when perturbation did not cover the onset + vowel or vowel + coda section as intended. Participants who had less than 16 trials in the hold phase with accurate location of the perturbation were removed from further calculations. Linear regressions confirmed no effect of number of trials on compensation magnitude after removing the failure trials. Based on this exclusion criterion, 34 participants remained in the Onset condition and 33 participants in the Coda condition (as reported in Oschkinat and Hoole, 2020).

#### Responses to temporally perturbed auditory feedback

The boxplots in Figure 2B (and the corresponding analyses performed in Oschkinat and Hoole, 2020) indicate that in the Onset condition, speakers did not change productions of the CC segment, but compensated for the vowel perturbation by lengthening the vowel in production. In the Coda condition, speakers compensated for the vowel perturbation by shortening the vowel, and for the Coda CC perturbation by lengthening Coda CC in production. The transitions from the Hold to the After-effect phase in Figure 2A indicate that in the Onset condition, the lengthening of the vowel (left panel, blue solid dots) was mainly an online response, as reactions revert to baseline shortly after perturbation is removed in the After-effect phase. The responses in the Coda condition (right panel, solid dots), on the other hand, were both indicative of adaptive behavior, visible in continuing adjustive responses from the Hold to the After-effect phase for both segments of interest (for statistics please refer to Oschkinat and Hoole, 2020).

The temporal adjustments in the Hold phase relative to the baseline mean (visualized in Figure 2B) will be taken as the measure for *compensation* to the perturbation per condition and segment. Note that the Onset CC and the Coda V were stretched in perturbation so that an opposing reaction is indicated by a shortening of productions (negative estimates relative to the baseline mean, see Figure 2). Hence, an opposing response to Onset CC and Coda V is necessarily adaptive. For the analyses in the current study, the values of the Onset CC responses and the Coda V responses were multiplied by –1. Thus, an opposing response is always indicated by a positive value and following the perturbation direction by a negative value.

Before turning to the motor and perceptual tests that will be related to the perturbation response patterns, we introduce here some brief analysis not included in the original Oschkinat and Hoole (2020) paper. Its purpose is to give additional preliminary motivation that consideration of individual behavior patterns should be fruitful by examining linear relationships between the four compensation measures introduced above. Conceptually, it would belong better with the motivations for the current investigation considered in the introduction, but can only be succinctly presented now that the reader has been given detailed information on the design of the previous experiment. Linear models were calculated between the four compensation measures (Onset CC, Onset V, Coda V, and Coda CC, with the outcome visualized in Table 1). The analyses revealed a significant linear relationship between Coda V and Coda CC (adjusted *R*-squared = 0.10, df = 31, *p* = 0.04), revealing that speakers who compensated more for the vowel perturbation in the Coda condition (by shortening it in production) also compensated somewhat more for the Coda CC segment (by lengthening it in production). Regarding our hypothesis, we assume that for these segments, which both showed adaptive behavior, a certain level of motor malleability is given in speakers that compensate and adapt more. However, since both segments appeared within the same word/trial, the magnitude of perturbation might have contributed to equally strong within-participant responses. A relationship was also found between Onset V and Coda CC responses, which both were second segments in the perturbation section, hence compressed in the auditory feedback but lengthened in production (adjusted *R*-squared = 0.196, df = 25, *p* = 0.01). Further, both segments were displaced in time, due to the stretch of the first segment and assumable subject to online control effects triggered by the stretch of the previous segment. The relationship supports the hypothesis that there might be an individual auditory sensitivity in speakers to react to effects of delayed/shifted auditory feedback in the online control. Both relations reinforce the aim of the current study to find individual motor or auditory abilities that enhance or decrease articulatory timing malleability in the face of an auditory perturbation, and more generally in the speech production process.

**TABLE 1:** Correlation table providing the adjusted *R*-squared and *p*-values for the relationships of the four compensation measures (compensation in the hold phase relative to baseline for Onset CC, Onset V, Coda V, Coda CC).

	Onset_V		Coda_V		Coda_CC	
	Adj. *R*^2^	*P*-value	Adj. R^2^	*P*-value	Adj. *R*^2^	*P*-value
Onset_CC	–0.026	0.658	0.051	0.135	–0.024	0.542
Onset_V			–0.04	0.99	**0.196**	**0.012**
Coda_V					**0.101**	**0.040**

Significant relations in bold.

### Tapping battery

For the tapping test block, participants were seated in front of a Roland SPD-6 MIDI percussion pad linked via a Midi-Interface (Miditech, midiface, 4 × 4) to a computer controlled by MAX-MSP software (version 6.0). Loudspeakers delivered sound stimuli in free field at a fixed volume which was kept constant over participants. The experimenter instructed the participant to tap with their writing hand’s index finger on the tapping pad. Practice trials preceded each of the following tasks, which could optionally be skipped when the following task was very similar to the preceding one. Tasks 1, 2, and 3 are adopted from the *Battery for the Assessment of Auditory Sensorimotor and Timing Abilities* (BAASTA, Dalla Bella et al., 2017). Tasks 4, 5, and 6 contain speech stimuli of different complexity implemented for this study’s particular purposes. All tapping tasks required the participants to tap as regularly as possible without intended variation in inter-beat interval or tempo. Except for the unpaced tapping task, all tasks differed in stimulus and inter-onset-interval (IOI, or inter-beat-interval, IBI, in music stimuli) of the respective stimulus beat. Since not much is known about the connection between finger tapping tasks and responses to temporal auditory feedback perturbation, a spectrum of different tests should provide insight into the connection between motor execution performance in different rhythmic contexts. The unpaced tapping task (Task 1) captures internal timing mechanisms. The metronome tapping tasks (Task 2) test for synchronization with a stimulus comprising a sequence of tones (metronome), the music tasks (Task 3) and speech tasks (Tasks 4–6) test beat detection in more complex stimuli. Thereby, the sentence tapping and music tapping require an identification of the beat in continuous sound flow, the syllable and wordlist tapping contain silence between each beat (here: word or syllable) analogously to the metronome tasks. The two music tasks differ in complexity. These tasks might provide further insight into timing abilities in different domains (speech/music). The single tapping tasks are summarized in Table 2, which gives a short explanation of the stimuli and the tempi performed by each participant.

**TABLE 2:** Overview of the performed finger tapping tasks.

Task Name	Tempo (ms)	Task/Explanation
(1) Unpaced	free	Regular Tapping for 60 s at a self-chosen tempo.
(2) Metronome	IOI 600, IOI 750 IOI 900	Tapping to a metronome (i.e., a sequence of tones with a frequency of 1319 Hz) for 60 s per tempo.
(3) Music	Rossini: IBI 600, Bach: IBI 600	Tapping to piano midi stimuli created from well-formed (regular) excerpts of the beginning of Bach’s *Badinerie* and Rossini’s *Wilhelm Tell*.
(4) Syllable	IOI 750	Tapping to the syllable “bla.” Four instances of the syllable “bla,” uttered by a German female speaker were randomly concatenated for 45 s with the IOI measured between the syllables’ supposed p-centers (determined using the algorithm from Cummins and Port, 1998).
(5) Wordlist	IOI 900	Tapping to a spoken wordlist (recorded by a female German speaker) of real monosyllabic words (nouns and adjectives) with complex onsets [CCV(C)]. Words were concatenated for 55 s with the IOI measured from the supposed p-centers (Cummins and Port, 1998).
(6) Sentence	IOI 600	Tapping to short sentences for 45 s (arranged from stimuli taken from Falk et al., 2017), repeated three times. Sentences presented a regular alternating rhythm (one unstressed – one stressed syllable) with an inter-stress-interval of 600 ms measured from the supposed p-center of each stressed syllable suggesting tapping on every second syllable.

All tapping data were pre-processed following the procedures as reported in Dalla Bella et al. (2017). The first ten taps were discarded in all tapping tasks, and artifacts (inter-tap intervals below 100 ms) and outliers were removed. For all tasks, including the unpaced tapping task, the mean inter-tap-interval (ITI) was calculated, and the coefficient of variation of the ITI (cv of the ITI, namely, the ratio of the standard deviation of the ITIs over the mean ITI) was taken as a measure for *motor variability*.

### Perception tasks

The third block tested for individual perceptual abilities. Five adaptive staircase tasks assessed individual auditory acuity performances for temporal properties of various stimulus types. The listener was seated in front of a computer and provided with headphones. Volume was set to a comfortable level as tested and determined by the experimenter and was not changed between listeners unless requested. After the experimenter started the procedure in MATLAB, listeners performed the tasks by entering their responses directly into the testing computer. The first three staircase tasks captured duration discrimination abilities (hence duration-based timing mechanisms) in a 2-interval 2-alternative forced-choice paradigm. These tasks required judgments about the two perceived stimuli as identical or different. Task 1 required judgments about pure tone durations. Tasks two and three comprised monosyllabic words with temporal manipulations analogous to the auditory feedback perturbation paradigm described in section “Temporal auditory feedback perturbation.” In the onset perception task, the onset of a word was stretched and the vowel compressed. In the coda perception task, the vowel was stretched and the coda compressed (see Table 3 for details). With these tasks an opportunity was provided to measure individual perceptual acuity of manipulated sound durations within a syllable similar to the auditory feedback perturbation. In addition to the three discrimination tasks, two beat-alignment tasks (BAT) related to the sentence and music tasks (Tasks 5 and 6) of the tapping battery in section “Tapping battery” were performed. Tasks 4 and 5 required beat-alignment judgments (and therefore event-based timing mechanisms) in a 1-interval 2-alternative forced-choice paradigm. The decision required a binary judgment on whether the metronome superimposed onto the auditory stimulus was aligned with the accents/beats of the speech or music stimuli or whether it was regular but shifted away from the natural accent/beat.

**TABLE 3:** Overview of the performed perception tasks.

Task Name	Stimuli/Continuum	Design/Task Question
(1) Pure Tone	Stimulus: Two pure tones (frequency: 333.3 Hz) Continuum endpoints: (1) Tone duration of 600 ms (2) Tone duration of 1200 ms	Design: 2-interval 2-alternative forced choice duration discrimination task Question: Do both tones have the same duration or not?
(2) Onset	Stimulus: Monosyllabic CVC word Continuum endpoints: (1) “Schaf” (/∫a:f/, sheep) and (2) “Schaf” manipulated, with /∫/ stretched by 200 ms and the following /a:/ compressed by 200 ms	Design: 2-interval-2-alternative forced choice duration discrimination task Question: Are the two words identical or different?
(3) Coda	Stimulus: Monosyllabic CVC word Continuum endpoints: (1) “Gas” (/ga:s/, gas) (2) “Gas” manipulated, with /a:/ stretched by 150 ms and the following /s/ compressed by 150 ms	Design: 2-interval-2-alternative forced choice duration discrimination task Question: Are the two words identical or different?
(4) Speech	Stimulus: Sentence with a metronome beat on every second (hence on every stressed) syllable at a stable tempo of 600 ms inter-beat-interval repeated three times (stimuli from Falk et al., 2017) Continuum endpoints: (1) Well-aligned metronome beat on every stressed syllable based on the p-center algorithm from Cummins and Port (1998) (2) Misplacement of the metronome beat by shifting it 200 ms later than in the original stimulus	Design: 1-interval-2-alternative forced choice beat-alignment task Question: Does the metronome match the stimulus or not?
(5) Music	Stimulus: Midi excerpt of Bach’s Badinerie (taken from Dalla Bella et al., 2017) Continuum endpoints: (1) Perfectly aligned metronome beat (2) Misplacement of the metronome beat by shifting it 200 ms later than in the original stimulus	Design: 1-interval-2-alternative forced choice beat-alignment task Question: Does the metronome match the stimulus or not?

For all five tasks, continua of stimuli between two endpoints were generated, whereby one endpoint consisted of the original stimulus and the second endpoint was a manipulated version. The manipulations target durations exclusively. For the three duration discrimination tasks (1–3), stimuli were presented in pairs (2-interval) in which one stimulus was always the original stimulus and the other stimulus varied in degree of manipulation between the two endpoints. Manipulations of segment durations were performed in Praat using *psola* (see task-specific description below). The presented stimuli were randomized, whereby the original stimulus was either in the first or in the second position. In the two beat-alignment tasks (4 and 5), one endpoint of the continuum was a stimulus with perfect beat-alignment, and the other endpoint a stimulus with the maximally shifted beat. In these tasks, always one stimulus from the continuum was presented while the degree of shift in alignment varied along the continuum. The difference between the two presented stimuli in Tasks 1, 2, and 3, or the degree of metronome shift in Tasks 4 and 5 is referred to as *delta* (in ms). In each task, the delta could be varied in increments of 1 ms. The maximum/start delta defines the largest difference between the stimuli or between the metronome and the stimulus. Estimations of the lowest correctly identified delta (based on calculations described further below) will serve to measure each participant’s individual auditory acuity.

**FIGURE 3:**
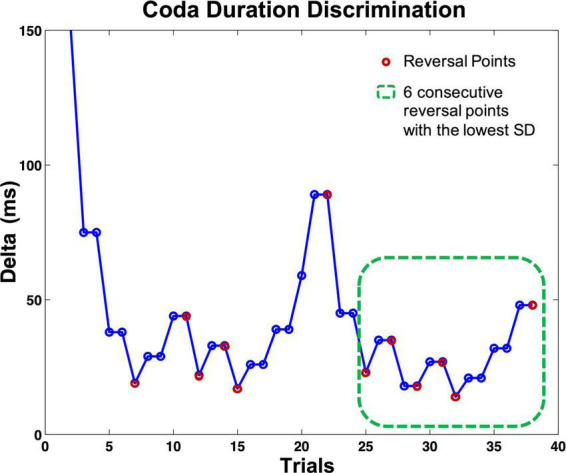
Visualization of the course of a perception staircase test (here: coda duration discrimination task). Circles indicate one presented stimuli pair. If the listener identified a pair correctly, the same pair was presented again, whereby the order of the two stimuli was randomized. After responding to the second presentation correctly, the delta of the following stimuli pair dropped; with every (single) wrong answer, the delta increased. The test ended after 12 reversals; each reversal point marked with a magenta circle. The green dashed square indicates the six consecutive reversal points (counting on from the reversal point at trial 25) with the lowest SD; the mean delta of these trials served as an auditory acuity score for further calculations. Catch-trials are not included in the figure.

All five staircase tasks had fixed but adaptive step intervals. In other terms, the next presented stimulus pair was triggered by the listener’s response. The tasks always required two correct difference detections in a row to the same stimuli pair to mark a successful identification, but only one incorrect response to mark a false identification (2 down/1 up protocol). The two stimuli in both presented trials appeared in random order. Following a successful mismatch identification, the current delta was multiplied by 0.5; with every not detected mismatch, the delta was multiplied by 1.5 (see Figure 3). Whenever there was a change of response quality (successful identification/false identification), one reversal was counted (see Figure 3). Each task ended when a fixed number of reversals was reached (12 reversals for the discrimination tasks 1–3, eight reversals for the beat-alignment tasks 4 and 5). Each task contained four to six presentations of two identical stimuli or a perfectly aligned beat (*catch-trials*). Listeners who did not identify more than 50% of the presented catch-trials correctly or did not reach a score below 70% of the start delta of a test were suspected of answering by chance or classified as incapable of performing the task. However, none of the participants had to be excluded from further calculations based on these criteria. To measure individual performance, for every participant and task, an individual auditory acuity score (i.e., a differential threshold) was assessed by calculating a mean over the delta of the most stable consecutive six reversal points in each task (the six reversals with the lowest SD, indicating a stable pattern of response), visualized in Figure 3. The most stable consecutive six reversals per test were chosen analogously to the procedure in Brunner et al. (2011).

## Analyses

The data provided by the Tapping and Perception blocks capture many motor and perceptual abilities for rhythmic speech and non-speech tasks. The following sections aim at summarizing the data, since the single tasks per block are expected to be highly collinear. In perception, the expected underlying mechanisms of the tasks as event-based or duration-based timing provide motivation to group tasks together based on timing mechanisms. However, it is also possible that dividing the tasks into speech and non-speech tasks reduces collinearity to a greater extent and explains the largest variance in the dataset. Further, in tapping, unpaced tapping can be accomplished successfully by engaging either beat-based mechanisms (i.e., generating an underlying pulse) or duration-based mechanisms (i.e., repetition of a single interval, Teki et al., 2011). Therefore, to reduce the complexity of the measures and find the most important dimensions in the data, we conduct one manual split: The unpaced tapping task will be treated as a separate measure, since it is the only tapping task that gives insight into pure feedforward timing mechanisms against eight paced tapping tasks. Further than that, principal component analyses for the (remaining) tapping and perception tasks are conducted. Summarizing the individual tasks per block into principal components should provide a mean performance measure per participant over tests that are highly correlative. In doing so, the responsibility for grouping the data in a meaningful way is passed on to the PCA. If there are substantial differences between the single tasks per block, they are expected to turn out as different underlying dimensions in the principal component analysis.

### Principal component analysis

The perception and tapping data were submitted to principal component analysis (PCA) using R’s *mclust* package (Scrucca et al., 2016). The PCA reduces the number of independent variables to single components by extracting the underlying dimension for variables that highly correlate with each other. The extracted underlying dimensions (principal components) of a PCA do not correlate with each other and describe the dataset’s maximum variance. In the following, the main components are extracted and used for further calculations. PCAs were calculated separately for all perceptual tasks (auditory acuity measures) and for all the tapping tasks (motor variability measures), except for the unpaced tapping task. The unpaced tapping task differed from the other tapping tasks in modality, as it was the only task without a pacing event. It gives insight into intrinsic motor timing without a guiding stimulus. Motor variability of the unpaced tapping task was individually taken into account in addition to the principal components. Unpaced tapping Motor variability was *z*-normalized before calculations. With this data partitioning, we hoped to keep one general underlying dimension per measure of interest (Perception, paced Tapping, unpaced Tapping) and further expected the PCA to give more insight into the nuances of the individual tasks.

#### Data pre-processing

As PCAs cannot deal with incomplete data, participants with no data in more than one out of the nine tapping tasks (including unpaced tapping) were not submitted to the PCA. Based on this criterion, five of 45 participants were removed before submitting the data to the PCA. For those participants who had missing data in one task, the missing value was filled with the k-nearest-neighbor imputation method (knn-imputation, Beretta and Santaniello, 2016). For one participant a missing value was imputed in the music Badinerie tapping task, for another participant in the metronome IOI 900 tapping task. Although not submitted to the PCA, this procedure was also applied to the unpaced tapping task. Two missing values in unpaced tapping were filled per knn-imputation.

Before submitting the paced tapping tasks to the PCA, the Kaiser–Meyer–Olkin measure (Kaiser, 1970) verified the measure sampling adequacy (MSA) overall per measure block and single task. The measure represents the *ratio of the squared correlation* between the single tasks to the *squared partial correlation* between the tasks. An MSA value of 0 indicates that the sum of partial correlations is large relative to the sum of correlations, suggesting too much diffusion in the data for factor analysis/PCA. An MSA of 1 indicates that the patterns of correlation are compact, indicating that the factors can distinguish the data reliably (Field et al., 2012, p. 769). An MSA value above 0.5 qualified the single measures for submitting them to the PCA, and the overall MSA measure classified the whole task block as suited for PCA if the overall MSA was > 0.5. In tapping motor variability, overall MSA was 0.68. However, the single MSAs for the Badinerie and Rossini music tapping tasks and the wordlist tapping task were < 0.5 and hence not submitted to the PCA. One could potentially leave these tasks aside and analyze them separately. However, this study aimed for an approach that optimized the coherence of the study as a whole, so these tasks were excluded from further calculations.

The same outlier and knn-imputation treatment as for the tapping data was applied to the perception data. However, no participant had missing values in the perception data. Hence no participant was removed and no data filled with knn-imputation. For the perception tasks, the overall MSA was 0.58 and all five tasks were kept in the PCA. Values were centered and scaled when submitted to the PCA.

**FIGURE 4:**
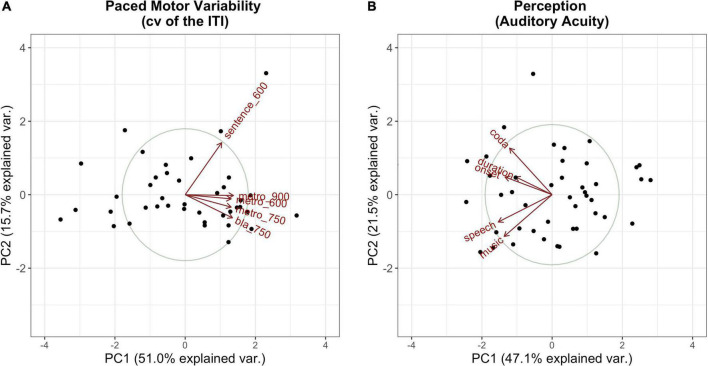
Visualization of the first (*x*-axis) and the second (*y*-axis) principal components along with their amount of explained variance per PCA (**A**: Tapping PCA, **B**: Perception PCA). Dots mark the single participants; vectors represent the factor loadings of the single tasks on each of the components. Tasks are abbreviated by type (e.g., “metro” for metronome tapping) followed by the IOI (if relevant).

The PCs that explained the most variance, whereby a substantial amount of explained variance is given for PCs with an eigenvalue > 1 (”Kaiser criterion,” Kaiser, 1960), were kept for further calculations. Those comprised the first principal component for the Tapping PCA and the first two principal components for the Perception PCA. Hence, PC2 in Tapping, which separates the sentence tapping from the metronome and syllable tapping, was dropped for further calculations. Figure 4 visualizes the first two components for each of the measures of interest.

#### Interpreting PC scores

Tables 4, 5 summarize the factor loadings of PC1 (and PC2) per PCA for each of the single tasks. Variables that have a larger loading than would be the case if all variables contributed equally, namely square root of 1 divided by the number of variables, will be regarded as important contributors to the respective principal component. For the Tapping PCA (Table 4), all presented tasks show fairly similar loadings on PC1. PC1 is therefore interpreted as a general measure for paced tapping motor variability. Correlation plots indicated the relation between the raw values submitted to the PCA and the PCs provided by the PCA. For *PC1 Paced Motor Variability*, better performances, meaning low motor variability values, are associated with lower PC1-scores. The same directionality applies to *Unpaced Tapping Motor Variability*, whereby lower values indicate low motor variability (hence a better performance).

**TABLE 4:** Factor loadings for each of the tapping tasks on PC1 for Motor Variability.

Task	PC1 paced motor variability
metro_600	0.46
metro_750	0.46
music_badine	–
music_ross	–
metro_900	0.48
bla_750	0.47
sentence_600	0.36
wordlist_900	–

High factor loadings on a component (|value| > 0.45) are shaded in grey.

**TABLE 5:** Factor loadings for each of the perception tasks on the PCs for the Perception PCA.

Task	PC1 perception	PC2 perception
Onset	–0.46	0.24
Coda	–0.41	0.64
Duration	–0.36	0.25
BAT speech	–0.52	–0.38
BAT music	–0.47	–0.57

High factor loadings on a component (|value| > 0.45) are shaded in gray.

For the Perception PCs, all of the tasks correlate negatively with PC1, with a higher PC score indicating a better perception (meaning a lower auditory acuity threshold). Therefore, PC1 reflects general auditory acuity and will further be referred to as *PC1 Auditory Acuity*. The music BAT perception task correlates negatively with PC2, as does the speech BAT task, although less intensely. The coda discrimination task correlates positively with PC2, and so do the onset and duration discrimination tasks, but less intensely. This clustering indicates that PC2 distinguishes beat-alignment judgments (event-based timing mechanisms) in speech and music from duration discrimination (duration-based timing mechanisms) and will further be named *PC2 BAT Perception*. Hereby, higher (positive) PC-scores are associated with better beat-alignment perception (especially in music) but worse duration discrimination (especially in the coda task). In view of this interpretation of PC2 BAT Perception, we expect this measure to be more closely connected to compensation in onsets, since we previously assumed onsets to rely more on event-based timing mechanisms. Duration-based perception tasks might be more closely coupled with compensation in the coda, since coda timing requires more likely absolute estimations of the time-lag from the onsets. Concerning the vowels, we have no precise hypothesis, since in the transition from onset to the vowel the p-center might play a role. The end of the vowel, on the other hand, might be more likely estimated with an absolute (duration-based) timing mechanism. The perturbation data and predictors provided by the Tapping and Perception blocks are summarized in Table 6.

**TABLE 6:** Overview of the measures of each of the three testing blocks along with the interpretation of the single PCs from the principal component analyses.

Test block	Quality	Measure 1	Measure 2
Perception	Auditory Acuity	PC1: Auditory Acuity	PC2: Beat-alignment (BAT) Perception
Tapping	Motor Variability	PC1: Paced Motor Variability	Unpaced Motor Variability
Perturbation	Onset compensation	Onset CC compensation	Onset V compensation
	Coda compensation	Coda V compensation	Coda CC compensation

The four perturbation measures (shaded in gray) will, due to their difference in articulation, position within the syllable, and perturbation direction, always be treated as different dependent variables. Measures 1 and 2 from Tapping and Perception will serve as predictors in model fitting.

### Outlier treatment (summary)

From the complete set of 45 participants in the beginning, the predominant basis for participant exclusion came from the perturbation data. Thirty-four participants remained in the Onset condition and 33 in the Coda condition after scanning the data for correct triggering of the perturbation (see section “Analyses”). The full set of participants was submitted to the PCAs for Tapping and Perception to get more reliable scores based on a larger dataset. Excluded from the full set were participants who had missing values in more than one of the tasks (five participants in Tapping, none for Perception), while data was imputed in the Tapping block for missing data in maximally one task per participant (applying to two participants). Data was also imputed for the Unpaced Tapping task (for two participants).

After calculating the PCAs, outliers of the generated principal components and the unpaced tapping task (data outside the 95% confidence intervals) were removed to reduce noise in the data. The same outlier treatment was applied to the perturbation response data.

The data were scanned for missing values based on outlier exclusion in the four Tapping/Perception measures (PC1 Auditory Acuity, PC1 BAT Perception, PC1 Paced Motor Variability, Unpaced Motor Variability). No participant had more than one missing value in the data. The single missing values were replaced with knn-imputation as performed on the raw data prior to the PCA (one participant: PC1 Auditory Acuity, two participants: Unpaced Motor Variability). Since the perturbation response data serve as the dependent variable in this study, none of the missing values (excluded participants as described in section “Temporal auditory feedback perturbation” and outliers) were imputed for the perturbation measures. The data was then divided into four datasets, each comprising one perturbation measure as the dependent variable (Onset CC, Onset V, Coda V, Coda CC) and the four Tapping/Perception measures as predictors (see Table 6 for an overview of measures).

After data exclusion and imputation, the remaining data comprised 28 participants for Onset CC perturbation, 29 for Onset V perturbation, 28 for Coda V perturbation, and 26 for Coda CC perturbation. Note that in visual presentation, outliers and imputed data after calculation of the PCAs are included but marked as such.

## Statistical modeling and results

The preceding section prepared the data for the following statistical analyses. These aim at testing our two main hypotheses that we firstly expect better auditory acuity and higher motor variability to be connected with more compensation. Secondly, we expect the perception measures to be more relevant for predicting effects in the online control, present in the Onset V and Coda CC, which were the second perturbed segments per condition. Further, we expect motor variability to be more connected with segments that were adapted for due to the perturbation, which was found in both segments in the Coda condition (Coda V and Coda CC). After interpreting the PCs in section “Interpreting PC scores,” we further assumed the PC2 BAT Perception to be more related to Onset CC compensation, since syllable onset timing in speech production has been suggested to rely on event-based timing mechanisms. However, since we did not find a significant effect of compensation for the Onset CC in the first place, this effect might not show in the analyses. We do not have a precise hypothesis regarding timing mechanisms of the tapping tasks, since the distinction is less pronounced than for the perception block.

To examine the most relevant predictors for responses per perturbed segment (CC or V) and perturbation condition (Onset vs. Coda condition), we make use of a machine learning technique by fitting regression trees to the data. Regression trees should provide insight into the most relevant predictors for splitting the data. Subsequently, linear models are fitted to the data with the predictors provided by the regression trees including their interactions to examine how well these predictors describe the variance in the data. In doing so, the regression tree analysis can be seen as an exploratory approach used for describing the most prominent qualitative features in the data. The linear models are then used as a confirmatory analysis to assess the robustness of the subdivisions into groups and provide the explained variance and statistical significance.

### Regression trees

Classification and Regression trees (CART, Breiman et al., 1984) are forms of decision trees that divide a dataset into further subgroups based on given discrete (classification) or continuous (regression) predictors. For each (sub)group, a simple model is fitted to predict the average outcome of the dependent variable. CART models are represented as a binary tree, whereby at each split one predictor is chosen by an algorithm detecting the least modeling error. For the purpose of our study, we adapted the method to process a relatively small dataset (*n* ≈ 29) to extract the most salient splitting criteria (predictors) of our data and to further model linear relationships between the predictors and the response data as suggested by the tree. Regression trees were fitted with the *rpart* function using the *rpart* package by Therneau and Atkinson (2019). Trees were visualized with the *rpart.plot* function/package (Milborrow, 2021). With this approach, the most descriptive of the four given predictors were extracted, and overfitting of the data avoided, which could occur due to the large number of predictors compared to the number of observations. The *rpart* function applies automated 10-fold cross-validation when choosing the best splitting predictor at each splitting point (*node*) and therefore reduces the risk of overfitting the data. For each node, the variable and a threshold along this variable are chosen to reduce the variance in the child nodes. At each splitting point, the variables are scanned for the error between the predicted and the measured values, and squared to get the sum of squared errors (SSE). The lowest SSE defines the splitting variable and the splitting point within the variable. This approach has some similarities with previous studies in which participants were split into groups of better performers vs. worse performers for a given variable. For example, Ghosh et al. (2010) divided their participants into “low- and high-acuity groups,” based on their median for auditory and somatosensory acuity, as previously done in Perkell et al. (2004b). In contrast to a traditional median split, the tree in the CART procedure first helps to decide on the best variable for dividing the data into groups, and crucially also lets the function choose the best threshold for splitting the participants along this selected predictor.

Four regression trees were fitted (one to each dataset) with the perturbation response data (compensation) as the dependent variable and PC1 Auditory Acuity, PC2 BAT Perception, PC1 Paced Motor Variability, and Unpaced Motor Variability as predictors, setting rpart’s *method* parameter to *anova*. The minimal number of participants for each split was set to four (*minsplit*), including the final splits (*minbucket*). Further, a cost complexity parameter (*cp*) has to be set to define the complexity of the tree. The *cp* decisively shapes the complexity of the tree and is a tuning parameter that should provide the best tree for predicting future data (balancing over- and underfitting of the tree model). A cp of 0 fits the most complex tree by predicting each observation (overfitting); a large *cp* might reduce the cross-validation error but increases the relative error and might underfit the data. To avoid underfitting of the small dataset, the *cp* per model was chosen based on the cross-validation error, but with a slightly greater tolerance toward a greater cross-validation error than in machine learning approaches. This approach was further motivated by the fact that in our case finding relationships in the data is of higher priority than actually predicting future data. Overfitting, in turn, was avoided by suppressing recursive splits of the same predictors that lead to a non-linear relation between the predictor and the outcome variable (*cp* per model: Onset CC: 0.1, Onset V: 0.15, Coda V: 0.15, Coda CC 0.1).^1^

**FIGURE 5:**
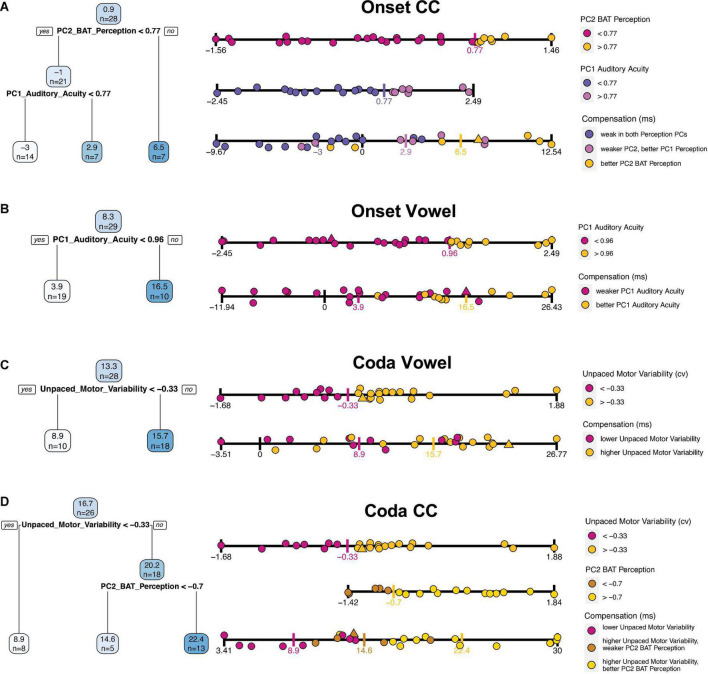
Fitted regression trees for each of the individual compensation measures in blue on the left side [from top to bottom: **(A)** Onset CC, **(B)** Onset Vowel, **(C)** Coda Vowel, **(D)** Coda CC]. Blue boxes are nodes and show predicted average compensation values at each splitting point, followed by the number of included participants. Darker blue boxes indicate higher compensation values, lower compensation values in lighter color. Below the blue boxes the variable that splits the data, along with an operator (> or <) and the estimated threshold for splitting. Participants who fulfill this criterion are included into the left branch (“yes”), participants who do not are assigned to the right branch (“no”). On the right next to the regression trees a further visualization of the splitting includes each participant’s performance. Each strip displays the variable according to the legend on the right next to it. The upper strips are splitting predictors; single participants are colored based on the thresholds as provided by the tree (threshold marked with a colored vertical tick). The lowest strip per plot displays compensation values, including the participants color-coded by the splitting thresholds per predictor variable. Triangles are imputed data for the respective predictor (upper strips), or of at least one of the predictors in the lowest (compensation) strip. Recall that lower motor variability corresponds to better performance in finger tapping.

The four tree models are presented in Figure 5. Per tree, each blue box shows an average of the outcome variable (compensation) and the number of participants that fall into this category/split. Below the first box, the variable is presented that first splits the data into two subgroups, along with the estimated threshold that splits the data along this variable. Participants who fulfill this criterion (dependent on the operator are above or below this threshold) are assigned to the branch on the left side (“yes”), those who do not are assigned to the branch on the right side (“no”).

### Interpreting regression trees

The quality of the predictions will be examined in turn for each of the four perturbed segments by looking at the tree models individually, supported by a more detailed visualization of each tree in Figure 5 using the strip plots on the right. While the upper strip(s) visualize partitioned data of the tree, the lowest strip visualizes the overlap of the groups by including all participants along the compensation scale. In particular, quite an informative impression of the prediction quality can be gained by looking at the extent to which the groups defined by the regression tree overlap.

The tree for Onset CC compensation shows the average amount of compensation (0.9%) for 28 participants in the blue box on the top (Figure 5A). The first splitting parameter is PC2 BAT Perception; this splits the dataset into participants with a PC2 BAT Perception above 0.77 (right branch), who on average have a compensation value of 6.5%, this applying to seven participants. Those who have a PC2 BAT Perception score lower than 0.77 have a mean compensation value of -1%, which accounts for 21 participants. For these 21 participants, PC1 Auditory Acuity was chosen as the most important parameter to further split the data, whereby participants with a PC1 Auditory Acuity score higher than 0.77 had larger compensation values (mean 2.9%; seven participants) and those with a score below 0.77 compensated less, or even more likely followed the perturbation (mean −3%, 14 participants). In summary, participants with better perception of beat-alignment compensated more. Those who were less good at perception of beat-alignment but better in general auditory acuity compensated a bit less, and those who showed weak abilities in both Perception PCs compensated least, or followed the perturbation direction. Recall here that compensation necessarily means the response is adaptive, since an opposing response is realized by shortening the Onset CC in production. A following of the perturbation direction due to weak perceptual skills could be explained by the lack of precisely detecting the direction of the auditory shift or determining the directionality of a response that would counteract the perceived shift. The relationships are further visualized on the right-hand side of Figure 5. In Figure 5A, the third strip plot (compensation) allows the overlap of the strongest (yellow dots) and weakest (slateblue dots) compensators to be visualized: The group of high performers in perception of beat-alignment (yellow dots) shows almost no overlap with the group of low performers in perception of beat-alignment and with low auditory acuity (slateblue dots, lowest strip), indicating a good prediction of compensation based on the two Perception PCs.

For vowel compensation in the Onset condition, only one split was achieved, namely with PC1 Auditory Acuity (Figure 5B). Participants with a PC1 Auditory Acuity higher than 0.96 typically compensated more (mean 16%; 10 participants), while those with a PC1 Auditory Acuity score lower than 0.96 compensated less (mean 3.9%; 19 participants). Therefore, participants with better auditory acuity (PC1 Auditory Acuity) compensated more for the vowel in the Onset condition (see lower “Compensation” strip plot in Figure 5B). For strong compensatory responses (above ∼17%) high PC1 Auditory Acuity performance (yellow dots) is found, while for very weak compensators and followers (below ∼4% compensation) only low PC1 Auditory Acuity performance (magenta dots) is found. But in the mid-range of compensation substantial overlap of the perception groups is shown, indicating the limits on prediction accuracy. Note, however, that none of the participants with high auditory acuity actually followed the perturbation (i.e., compensation < 0).

For vowel compensation in the Coda condition (Figure 5C), only Unpaced Motor Variability emerged as a splitting factor, whereby participants with higher Unpaced Motor Variability (>–0.33) showed stronger compensatory responses (mean 15.7%; 18 participants), and participants with lower Unpaced Motor Variability compensated less (mean 8.9%; 10 participants). Overall prediction accuracy appears quite weak, since the two groups overlap substantially (“Compensation” strip plot, Figure 5C). Nonetheless, it seems worth pointing out that the nine participants with the strongest response (>∼17% compensation) belong to the high motor variability group (yellow dots).

Finally, the tree for CC compensation in the Coda Condition (Figure 5D) was first split with regard to Unpaced Motor Variability, whereby participants with Unpaced Motor Variability higher than -0.33 compensated more (mean 20.2%; 18 participants), and speakers below this score (less Unpaced Motor Variability) compensated less (mean 8.9%; 8 participants). For those with higher Unpaced Motor Variability (above –0.33), PC2 BAT Perception split the data further into subgroups, whereby participants with better perception of beat-alignment (>–0.7) compensated to a greater extent (mean 22.4%; 13 participants), while those with a lower perception score compensated less (mean 14.6%; five participants). To summarize, higher motor variability in unpaced tapping leads to stronger compensatory responses. For participants with higher motor variability, better beat-alignment perception abilities enhance compensatory responses and lower beat-alignment perception performance weakens responses. As visualized in the “Compensation” strip plot of Figure 5D, the group of high Unpaced Motor Variability and high PC2 BAT Perception performers (yellow dots) does not overlap (except one participant) with the group of low Unpaced Motor Variability performers (magenta dots), indicating quite precise prediction of compensation by the tree model. The in-between group with higher Unpaced Motor Variability and weaker perception of beat-alignment (brown dots) shows medium strong responses. This group selection supports the idea that better perceptual abilities lead to stronger compensation, but only if a certain malleability in the motor system is given.

In summary, the most relevant predictors for compensation in the Onset condition were the perception PCs, while for the Coda condition Unpaced Motor Variability was most relevant. Vowel prediction was generally less pronounced than CC prediction, as indicated by the overlap of the groups in the “Compensation” strips in Figure 5. The division into “good” and “worse” performers in Unpaced Motor Variability or perception was typically computed by dividing into thirds rather than at the median or mean along the respective variable. Note here that PC1 Paced Motor Variability did not achieve a split in the data for any segment/condition, and will therefore be dropped in the following analyses.

### Linear models

**FIGURE 6:**
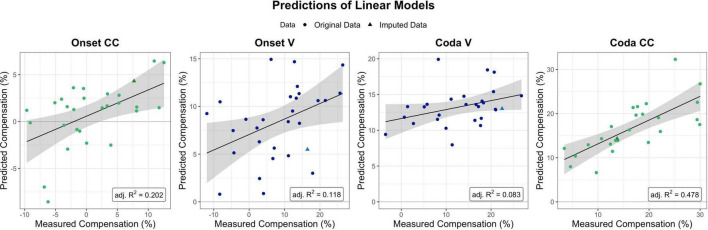
Predictions as provided by the linear models per segment and condition of interest (with the four perturbation measures ordered chronologically from Onset CC on the left to Coda CC on the right). Model fits for Onset CC, Onset V, and Coda CC are significant, Coda V non-significant. The Coda CC model was the one explaining most variance in the data (adj. *R*-squared = 0.478). Dots mark single participants, triangles single participants for which data was imputed for (at least one of) the predictors.

To further quantify the quality of the subdivisions achieved by the trees, linear models were fitted with the predictors derived from the regression tree analysis. Analyses were performed using the *lm* function of R’s included stats package (v4.1.2). These aimed at providing an indication of the accuracy of the modeled trees without a test and a training set, but by including the predictors provided by the respective regression tree and their interaction into a linear regression model. The prediction of each linear model, i.e., predicted compensation values versus measured compensation values, is visualized in Figure 6.

The linear model for CC compensation in the Onset condition was therefore modeled with compensation as the dependent variable, and both perception PCs and their interaction as predictors. The result indicated that the model was significant [*F*(3,24) = 3.28, *p* = 0.038] and explained 20.2% of the variance in the data (adjusted R-squared). The model revealed a significant contribution of PC2 BAT Perception to modeling the data (*t* = 2.15, *p* = 0.041), but no significant contribution of PC1 Auditory Acuity (*t* = 0.430, *p* = 0.670) nor the interaction between both Perception PCs (*t* = –1.624, *p* = 0.117).

The linear model for vowel compensation in the Onset condition was computed with compensation as the dependent variable and PC1 Auditory Acuity as predictor. The model explained 11.8% of the variance in the data and was significant, *F*(1,27) = 4.74, *p* = 0.038. The model revealed a significant contribution of PC1 Auditory Acuity to modeling the data (*t* = 2.178, *p* = 0.038).

Vowel compensation in the Coda condition was modeled with Unpaced Motor Variability as a predictor. Overall model fit was quite weak (adjusted R-squared = 0.083), and non-significant, *F*(1,26) = 3.44, *p* = 0.075.

CC compensation in the Coda condition was modeled with Unpaced Motor Variability and PC2 BAT Perception as predictors as well as their interaction. The model was significant, *F*(3,22) = 8.617, *p* < 0.001, and accounted for 47.8% of the variance. Unpaced Motor Variability contributed significantly to the model (*t* = 4.617, *p* < 0.001), and so did PC2 BAT Perception (*t* = 2.126, *p* = 0.045). The interaction between both predictors did not contribute significantly (*t* = 1.210, *p* = 0.239).

### Speech motor variability and compensation

**FIGURE 7:**
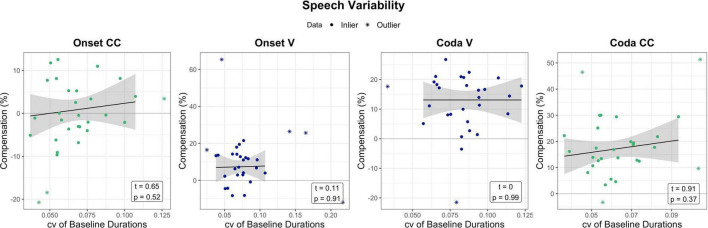
Relation between baseline variability in the perturbation experiment (coefficient of variation of baseline durations per segment) in relation to compensation. Effects are non-significant. Green and blue dots mark the data that has been used for model prediction. Stars are outliers that have been excluded in statistical modeling. The black line visualizes the regression of the data (dots) without outliers (stars).

While the previous section indicated that non-verbal motor abilities relate to responses to temporal auditory feedback perturbation, one could ask if similar effects can be seen for speech motor variability and perturbation. The following section briefly examines temporal speech variability in the baseline of the perturbation experiment and its relation to compensatory behavior. For the assessment of speech motor variability, data from the perturbation experiment was examined. The coefficient of variation (standard deviation divided by the mean) of the word-normalized segment durations (V and CC) produced in the baseline phase per experiment condition (Onset/Coda) per participant was calculated. The first nine trials of the baseline were not included into calculations, as they were excluded in analyses in Oschkinat and Hoole (2020). The coefficient of variation (cv) of baseline productions per segment and condition was then correlated with the respective compensation measure. Unlike tapping motor variability, the cv of the baseline segment durations were not significantly related to the amount of compensation. Figure 7 visualizes the relationship between compensation and baseline variability per segment of interest, accompanied by statistical outcome of the correlations.

## Discussion

### Main findings

The present study investigated the connection of individual perceptual and general motor abilities to responses to temporal auditory feedback perturbation. Our results support the idea that individual perceptual abilities and individual capacities in precise motor execution both shape the speech production process. The extracted qualities were summarized by their underlying dimensions obtained from a principal component analysis and served as predictors in statistical modeling. The analyses followed an exploratory-confirmatory approach: Regression trees selected the most relevant predictors, which subsequently were included in linear modeling. In model prediction, beat-alignment perception, general auditory acuity, and motor variability of an unpaced tapping task explained variance in the perturbation response data. In general, the perceptual dimension generated the most prominent predictors for describing variance in response to temporal onset perturbation of syllables (applying to both complex onsets and the following vowel). In contrast, motor variability of unpaced tapping was most relevant to predict responses to temporally perturbed auditory feedback of syllable codas (significant for consonant coda clusters, non-significant for the preceding vowel). This relationship supports our second hypothesis, suggesting that greater motor variability allows for more adaptation (whereby adaptation was only found in the Coda condition). Auditory acuity, on the other hand, was suggested to be relevant for responses in the online control as well as adaptation. Indeed, auditory acuity explained variance in compensation to the segments in the Onset condition, and the Coda CC in the Coda condition. Thereby, the vowel in the Onset condition and CC in the Coda condition were the second perturbed segment and therefore exposed to online effects induced by the stretching of the preceding segment. This result is in line with the findings by Martin et al. (2018), who found auditory acuity more relevant for online effects than for long-term adaptation of motor commands. The vowel in the Coda condition showed the weakest predictability, as the only segment that comprised exclusively adaptive responses and was compensatorily shortened in production. Better perceptual abilities and higher motor variability were linked to stronger compensation, as expected in our first hypothesis (but see the contribution of PC2 BAT Perception for Coda CC compensation discussed further below).

Concerning the motor variability component, there is, to our knowledge, until now no study that explicitly investigated the connection between individual motor timing capacities and responses to perturbed feedback. However, the current study’s results align with our main conclusion drawn from Oschkinat and Hoole (2020): Greater variability leads to a less stable system that is more malleable in the face of auditory feedback perturbation. In Oschkinat and Hoole (2020), this assumption was directed to *structural* effects: Syllable onsets are articulatorily more stable than syllable codas and therefore less malleable in the face of an auditory feedback perturbation. The data of the current study suggest that structurally given malleability can be further modulated by *individual* motor stability in temporal auditory feedback perturbation. Especially, perturbation to coda segments (the prediction accuracy of R-squared 0.47% was by far the highest of our four conditions) showed that low motor variability (better temporal stability) was linked to less compensatory responses, more precisely to less *adaptation*. This assumption is further supported by the correlation of the compensation measures in Coda condition with each other. Both segments seem to share a certain malleability, although this has to be interpreted carefully because they were also manipulated within one perturbation frame. For speakers with higher motor variability, better perceptual performance increased compensatory responses, and weaker perceptual performance weakened compensatory responses. This interplay indicates a tradeoff between perceptual and motor abilities, and supports the finding that better perceptual abilities do indeed lead to more compensation/adaptation, but that adaptation only occurs if a certain system malleability is given. For future paradigms that aim at capturing strong adaptive responses, it might be particularly revealing to focus on speakers with this specific combination of high auditory acuity and high motor variability, and provoke adaptive shortening responses rather than lengthening, since the latter might always contain not just an adaptive component but also an online response.

### Timing mechanisms

Regarding the perceptual components, these findings are in line with previous studies that examined the link between auditory acuity and responses to spectral feedback alterations. For example, in Villacorta et al. (2007) and Brunner et al. (2011), speakers with higher auditory acuity compensated more for a spectral shift in the auditory feedback of vowels. Still, the comparability with these studies is not naturally given: While Villacorta et al. (2007) assessed auditory discrimination ability for F1 when F1 was perturbed in the experiment, the perceptual correlate of perturbed speech timing is not self-evident (see Oschkinat and Hoole, 2022 for discussion). In our data, both duration discrimination and perceptual beat-alignment abilities were linked to temporal feedback perturbation. The regression tree structure for the Onset CC condition (Figure 5A) suggests that beat-alignment judgments (event-based timing mechanisms, represented by PC2 BAT Perception) are most relevant for predicting compensatory behavior for temporal perturbation of the complex onset. This relation supports our minor hypothesis raised after interpreting the PCs in section “Interpreting PC scores” that event-based timing mechanisms might be more relevant in predicting behavior in onsets. Speakers who more precisely detect a shift of the p-center in the auditory feedback (as introduced with the stretched Onset CC in perturbation), may adjust more for it. However, PC2 BAT Perception further explained variance in compensation to the Coda CC segment, which is not as expected or explainable with the p-center concept. In this case, recall that higher PC2 BAT Perception scores were further associated with weaker perceptual abilities in discriminating duration differences in codas (coda perception task). Therefore, the prediction of beat-alignment timing being more closely associated with onsets and duration-based timing with codas could not be fully supported. The Onset CC regression tree analysis further suggested that good duration discrimination abilities (duration-based timing, PC1 Auditory Acuity) can partially counteract worse PC2 BAT Perception abilities. Moreover, bad performance in both perception domains leads to poor compensation, or more precisely, mainly to a following of the perturbation (negative responses). The predictability of following responses from poor perceptual skills might result from the inability of speakers to precisely locate either the direction of the shift in the auditory feedback or the direction in response that would oppose the perceived shift direction. The present findings further add to the discussion about what leads to following responses in so many perturbation studies (see, e.g., Katseff et al., 2012; Franken et al., 2018). The aforementioned perceptual abilities explained 20.2% of the variance in our data (Onset CC condition). Of the substantial remaining variance, some of it might be explained, for example, by how speakers balance auditory against somatosensory errors (Katseff et al., 2012). Indeed, we have suggested that somatosensory feedback may be particularly relevant in syllable onsets, since somatosensory feedback is accessible earlier than auditory feedback. In syllable onsets, auditory feedback cannot be used to estimate relative durations within the syllable as it is possible in codas, where onset and vowel durations have been already perceived. Therefore, somatosensory feedback could be more informative for error correction in timing (Oschkinat and Hoole, 2020).

### Speech motor variability

While motor variability in unpaced tapping correlated with responses in the Coda condition, a similar relationship could not be found for measures of (temporal) *speech* motor variability. Certainly, these results need to be interpreted cautiously. While the unpaced tapping task tests pure, task-unspecific internal timing stability with a low-complexity motor task, speech production requires a complex coproduction of muscles, each of them allowing for variability/degrees of freedom in the execution. Further, speech variability measures were assessed from only 11 trials in the baseline phase, which might not give a solid mean for such analyses. Regarding the motor variability measure from finger tapping, we nevertheless admit that in using this measure as an indication for internal variability/malleability, we cannot precisely disentangle imprecision in motor execution from imprecision in the internal motor plan. However, PC1 Paced Motor Variability did not seem relevant in explaining the data of this study and was dropped as a predictor for compensation in the regression tree analyses. One might conclude here that the difference between paced and unpaced timing tasks is not the ability to precisely execute motor commands according to an internal plan, but rather the internal rhythmic representation that is needed in unpaced tapping but externally provided in paced tapping. By closely examining previous studies, the results turn out to be partially in line with investigations on spectral speech variability and compensation to spectral perturbation. In previous studies, compensation to spectral perturbations correlated with the variability of contrast of different speech sounds in production (Ghosh et al., 2010; Brunner et al., 2011). However, compensation did not correlate with the variability of one individual parameter (e.g., F1) in repeated phoneme productions (MacDonald et al., 2010, 2011). Mixed findings were also provided by Nault and Munhall (2020), who conducted a study on inter-and intraspeaker variability. They measured the standard deviation of the first two formants of vowels produced in the baseline phase of a spectral perturbation experiment and found a relation between F1 variability in the baseline and F1 compensation in the hold phase, but no contribution of baseline variability of F2 as a predictor for compensation to perturbed F2. Another recent study neither found relations between adaptation and vowel spacing in the baseline phase nor correlations between adaptation and variability in productions of single baseline phonemes (Parrell and Niziolek, 2021). In this view, the non-existent relationship between speech variability and compensation in our data is in line with the findings by MacDonald et al. (2010, 2011), Parrell and Niziolek (2021), and partially Nault and Munhall (2020). However, analogously to the variety of spectral measures, there is still considerable room to ponder about the best parameter to measure variability in production of speech timing. Further, it has to be kept in mind that temporal information of speech is different from spectral information: While spectral properties of fricatives and vowels serve to distinguish similar sounds from each other, duration’s primary purpose is not to distinguish sounds but to give their spectral evolvement a stage^2^. Individual variability in production might not be relevant when high variability cannot result in another category. The distinctive function of duration is much less pronounced than the distinctive function of spectral properties of speech. Duration and timing are certainly not arbitrary but follow different goals, such as enabling fluency and intelligibility and realizing prosodic aspects of speech.

## Conclusion

The findings of the current study gave insights into how feedback and feedforward mechanisms in speech and non-speech are connected and how their interaction shapes timing in speech production. We also believe that the study provides a valuable foundation for guiding future studies in the selection of more targeted perception and tapping tests for similar research approaches. In particular, motor variability in tapping tasks seems worth further exploration; especially unpaced tapping as a measure of general internal motor stability should be considered. Regarding the significance of the current and similar studies, it should be noted that to date not much is known about the reproducibility of reactions to (temporal) auditory feedback perturbation. Saying this, there is certainly a need for establishing a firm understanding of how compensatory responses to the same perturbations vary within participant across multiple sessions. Further, in future investigations, it might be worth looking into groups of participants with different levels of auditory acuity and motor stability, such as musicians and non-musicians. In follow-up investigations, the perception staircase paradigm could be improved by using a 4-interval 2-alternative AABA design, which would probably provide more reliable threshold estimations than the 2-interval paradigm with catch-trials or an ABX paradigm (Gerrits and Schouten, 2004). Finally, it should be noted that in our PCA approach, some tasks were dropped in calculations, and relationships between the single motor and perception tasks and compensation to temporal perturbation might have been blurred. Nevertheless, we see the PCA-driven analyses as clearly crucial here in making the large number of individual tasks tractable for analysis, and indeed provided interesting insights, e.g., by distinguishing perception tasks based on timing mechanisms.

## Data availability statement

The measurement data supporting the conclusions of this article will be made available by the authors, without undue reservation.

## Ethics statement

The studies involving human participants were reviewed and approved by the Ethics Committee of the Medical Faculty of the Ludwig Maximilian University. The participants provided their written informed consent to participate in this study.

## Author contributions

MO, PH, SF, and SDB: study design, data processing, and writing. MO: data acquisition and analyses. All authors contributed to the article and approved the submitted version.

## Footnotes

1 While we believe that non-linear relationships between auditory acuity and compensation, and particularly between motor variability and compensation are readily conceivable, substantially larger numbers of observations would be needed to fit and assess such models.

2 We are not, of course, ignoring the presence of quantity distinctions.

## Appendix

### Effects of musicality

Along with the three experiment blocks, musical education of the participants was assessed by a questionnaire asking about whether they received musical education, where they received it, for how long, on what instruments/singing, and whether they learned an instrument without education. Since musicality was not the main interest in this study, for a simple overview, participants were grouped into those who received musical education (at least for 2 years), and those who did not receive any musical education (non-musical group).

Additional analyses showed that when comparing the two groups with a two-sampled Welch test, no group differences were observed for PC1 Paced Motor Variability nor for Unpaced Tapping Motor Variability, PC1 Auditory Acuity nor PC2 BAT Perception (non-musical group: *n* = 10, musical group: *n* = 25). Note here, however, that the group of musically educated participants was much larger than the group of non-musically-educated participants, and that there was high variability in duration of musical education within the group of musicians (from 2 years up to 13 years on one instrument), and in the start age of musical education on the first instrument (5–13 years of age).

Further, there were no group differences in response to the temporal perturbation for the Onset CC (non-musical group: *n* = 9, musical group: *n* = 19), no difference for Onset V (non-musical group: *n* = 9, musical group: *n* = 20), and the Coda CC (non-musical group: *n* = 7, musical group: *n* = 19). For the Coda V, the group of musically educated participants adapted less than the non-musical group (*t* = 2.447, df = 15.99, *p*-value = 0.026, non-musical group: *n* = 8, musical group: *n* = 20). Since the Coda V was the only segment that exhibited significant adaptation effects, this connection suggests that musicians showed more resistance in adapting to perceived errors than non-musically trained participants. Note however, that this relationship was not found for Unpaced Motor Variability or PC1 Motor Variability, perhaps due to a different subset of participants based on outlier exclusion. The effect of musical training on adaptation should give a direction for future studies and suggests that highly trained musicians as compared to non-trained musicians might be a group worth investigating more closely.
